# Somalia Famine, another Predictable Disaster in the New Millennium

**DOI:** 10.5812/kowsar.20741804.2311

**Published:** 2011-09-15

**Authors:** K B Lankarani

**Affiliations:** 1 Health Policy Research Center, Shiraz University of Medical Sciences, Shiraz, Iran

**Keywords:** Somalia, Famine, Disaster

Somalia has been one of the most politically unstable countries in the past two decades. Having no central government since 19 years ago, the country has lost most of its infrastructures during the domestic wars. This year, the problem of drought and famine has worsened the condition even further. It is estimated that half of the population in Somalia are now suffering from food insecurity, near 10% of these are children.[1][2] There are at least 200,000 severely malnourished children in the need of immediate care. [1][2]A recent survey indicates a prevalence of global acute malnutrition of more than 20% in the most regions of Somalia.[3] Even the progresses that were reached for control of infectious diseases like measles through novel strategies by local authorities with the support of international partners now seems to be lost with near total destruction of health system.[4] Cases of visceral leishmaniasis, malaria, measles, respiratory and gastrointestinal tract infections are all escalating in number on a daily basis. [1][2]Iranian aid workers who were in the field in the camp established by Iran Red Crescent Society in Somalia report the condition is much worse. Deaths of infants, children and their mothers are constant events. Despite much propaganda, few things have been done in reality. The relief camps in Mogadishu, the capital can not respond to the needs with many shortcomings in logistics. Health workers are few and not prepared for such a disaster. The problem of war is still there. In some scenes, the armed militants even asked for money to secure of the relief camps and in some instances some of these groups even attacked the relief aids. The transitional government is weak with little authority to control the situation.

The severe famine and concomitant armed conflict have pushed many people to migrate to neighbor countries specially Kenya. Interestingly, the general condition of these refugees has become worse in the past month indicating more malnutrition with resultant higher mortality rate. Reaching these camps is not an easy task.[2] The trek takes almost 17 days of constant walking with little access to food and water. Many of the refugees specially the children and elderly died during this journey.[1] Those lucky ones who reached the camps, could not receive a good care as the food and water supply and the ability to provide medical care to these poor people were very limited there.

We can not ignore the effect of war and domestic violence in expanding the dimensions of this disaster. But the problem of famine is not restricted to war torn Somalia. Many are also affected in Kenya, Ethiopia and other countries.[1] In Somalia, most of the affected parts in south are under control of a militant group called Al Shabab who are believed to have close ties with Al Qaeda . Recently United States of America have intensified its attacks to this region both directly and indirectly through their local partners. These attacks mostly targeting heavy populated regions with most inhabitants being civilians that have made the situation even much more complex. This new war after two decades of instability in the country has been linked to the control of oil resources found in the Somalia.[5]

There has been criticism on the way the famine was handled by international organizations specially United Nation and its partners.[1] The donors also were criticized for their delayed response. Interestingly, the eyewitnesses in the region noted the food were available in the markets even in Somalia. But the problem is the high skyrocketing price of the food that has tripled in the past three months. Poverty and loss of the live stocks during the drought which were usually the fundamental fund of the families have left these families without any financial support. That is the reason why some advocates suggest in substitution of sending foods to the region in a complicated way and usually in insufficient amount, it might be more practical and even more effective to transfer cash to these countries and provide the families with financial support to buy their own needs from the domestic market. It should be mentioned that at least some of these foods in the market are wholesale of the theft food aid. [6]

Although most observers agree that the most effective solution is development in all aspects specially education and in establishment of infrastructures and stopping the war, all would also mention the necessity of an immediate action.

Looking behind, there is a concern whether the event could have been predicted? This drought in the Horn of Africa was forecasted in 2010 as a part of global climate hange.1 Unfortunately this forecasting was not taken seriously.[1] Even the increasing number of refugees as was reported by international relief agencies was not considered as a problem.[1][2] The result of this negligence is now felt painfully with fear of expansion of the problem to other countries.

The World Health Organization (WHO) has developed a global alert and response system mostly aimed for early alert on global threats from infectious diseases. [7] This system worked reasonably during the flu pandemic in 2009 and beyond. It seems with the growing problem of climate change, there is a need to be more collaboration between WHO and World Meteorological Organization (WMO) which is the specialized United Nation's Agency on weather and climate. It is now quite possible to predict disasters like the drought in eastern Africa months before its peak. These alarms and forecasts should be taken seriously and a global action planned. WHO could play a central role in this regard? Recently a joint project have been launched by WHO along with United Nation Development Program (UNDP) as a pilot program in seven countries including Kenya to increase adaptive capacity of national health systems to respond to climate sensitive health risks.[8] Although this could be a good start but the problem of climate change is more huge and more urgent to wait for developing and then scaling up of these pilot projects. Climate change would become worse and worse and events like the tragedy in the Horn of Africa would become more frequent. WHO can work as an alerting agent and bring the problems to the eyes before it reaches the irreversible stage. If world took the forecasting of this drought on 2010 seriously many lives could have been saved.
